# NASH: Connecting the dots with patients in mind

**DOI:** 10.1002/edm2.109

**Published:** 2020-10-21

**Authors:** Donna R. Cryer

**Affiliations:** ^1^ Global Liver Institute Washington District of Columbia

## Abstract

Liver patient, patient advocate and patient advocacy organization leader describe the requisite actions for connecting patients, physicians, policymakers and global leaders into a global movement to address the epidemic of nonalcoholic steatohepatitis.

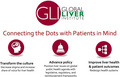

Throughout this special issue, other authors have described the challenges of connecting—connecting genotype to phenotype, biomarkers to biopsies, diagnostics to drugs, science to regulation. The role of patient advocacy in this field is also first and foremost a role of connector.

Nonalcoholic fatty liver disease (NAFLD) is one of the most common causes of chronic liver disease in the United States, affecting nearly 100 million individuals.^1^ If one were to diagnose the historic affliction of the liver community—undersized, undernourished relative to the overwhelming size and ferocity of liver diseases or compared with other disease communities, whether we look at HIV/AIDS or cancer, one would see a condition of disconnection. In the case of fatty liver disease and NASH, patients are not connecting the impact of their behaviour, their genetics and their metabolism to their liver health. Primary care physicians are not connecting the patients presenting with multiple aspects of the metabolic system to screening and treatment for liver disease. Hepatologists have not traditionally connected to diabetologists or lipid specialists in care settings to collaboratively manage patients. Policymakers are not connecting the rising rates of fatty liver disease and NASH alongside obesity, including childhood obesity, diabetes, heart disease and even some cancers in their communities to integrate NAFLD and NASH into policies to advance research, surveillance, prevention and care delivery. Finally, the global public health agenda‐setting bodies are have not been connected to liver advocates and issues in a comprehensive long‐term manner that creates both urgency and sustained structural and strategic approaches to combatting the fatty liver disease/NASH epidemic. To cure this condition, we need to connect the challenges of NAFLD/NASH community to the information, resources and relationships that can make us stronger.

As a patient, patient advocate and leader of a patient advocacy organization, I recognized the need to create a platform for connection in the GLI NASH Council which launched in October 2017 at the GW Milken Institute School of Public Health and has now grown to 44 member organizations—medical and nursing societies, academic NAFLD clinics, government consortia and industry partners.

## 1 CONNECTING THE DOTS FOR THE PATIENT

According to a survey conducted by one of the NASH Council members, 33% of the American public believes that they do not need a liver to survive.^2^ Most people cannot locate their liver if asked nor describe what it does beyond metabolizing alcohol. Patients frequently report feeling isolated, stigmatized and unable to share their diagnosis even with close friends and family. With no FDA or EMA—approved medication specifically for NASH, lifestyle change resulting in a weight loss of 7%‐10% of body weight is the primary mechanism for patients to stall or reverse disease progression. Extensive lifestyle change has the most likelihood for success in the context of support, personal, peer and professional. Connecting patients to their sense of self‐efficacy and their communities is essential to defeating NASH.

## 2 CONNECTING THE DOTS FOR PRIMARY CARE

The American College of Physicians welcomed leading hepatologist, Dr Zobair Younossi, to a prime speaking spot at their annual meeting in 2018, as a crucial step for internal medicine and family physicians to understand the NASH is their issue and an issue for their patients. Connecting hepatologists to nonhepatology conferences and meetings is a key measure of success for the NASH Council.

It is estimated that only a small proportion of NASH patients diagnosed and even fewer are biopsy‐confirmed. Yet the number of patients presenting in primary care with two or more risk factors for NAFLD or NASH—obesity, type 2 diabetes, high cholesterol and high triglycerides—is increasing.^3^ The number one request I get when speaking with internal medicine physicians or medical society staff is for actionable information for screening patients like A1C for diabetes or DEXA for osteoporosis that fits into current office workflow. Until biomarkers are validated against liver biopsy, and with the limitations of biopsy in‐sample error, cost and risk to patient, there is urgency around validating these alternatives, advances by centres integrating algorithms like the Fibrosis‐4 (FIB‐4) in their electronic health records can have significant impact in ensuring that fewer patients in the primary care setting remain undiagnosed and connected to appropriate management, as hepatitis C EHR‐based care cascades proved.

## 3 CONNECTING THE DOTS FOR SPECIALISTS

NASH, for most patients, is the hepatic manifestation of metabolic syndrome. The growing appreciation of this medical fact is compelling collaborations between hepatologists, endocrinologists and cardiologists often, for the first time, and influencing the update of guidelines for management of patients with fatty liver disease, diabetes and heart disease. Greater integration of NALFD/NASH into clinical workflows and treatment algorithms will necessitate new conversations on which specialty will take lead responsibility for patients, when, and at what disease activity thresholds. Patients with these concurrent diseases and patient advocates across these diseases and conditions will also need to work together and push for holistic, whole‐person management from physicians, health systems and payers.

## 4 CONNECTING THE DOTS FOR POLICYMAKERS

Working with the sole hepatologist in the US Congress, Sen. Bill Cassidy, GLI cohosted a Congressional briefing that featured a NASH patient and transplant recipient with speakers representing AASLD, AGA, ADA and the Endocrine Society. Members from the various caucuses— Cancer, Hepatitis, Diabetes, Public Health, Biomedical Research, Black, Hispanic and Asian Pacific Islander— and key Senate health committees were invited, representing the intersectionality of the condition. Many of the calls to action made by advocates that day have been picked up by the Liver Illness Visibility Education and Research Act of 2019 (HR 3016)^4^ which recognizes the link between liver diseases like NASH and cancer and multifaceted approach needed to fully address liver diseases sustainable.

## 5 CONNECTING THE DOTS FOR THE WORLD

Patient advocates in Europe launched the 1st NASH Summit in Parliament in 2017. In June 2019, in partnership with the Global Liver Institute under the International NASH Day banner, 65 partners in 22 countries, including 12 national patient organizations and foundations, 15 universities, hospitals and clinics, and several corporations conducted screenings, educational fora, a documentary film premiere, web, tv and social media activities to raise awareness of fatty liver disease and NASH. This affords the opportunity to establish a global infrastructure for culturally relevant community‐driven education, support and advocacy activities not only for NASH but for liver health initiatives.

In July 2019, the World Health Organization issued a call for countries to invest in the elimination of viral hepatitis utilizing the cost‐effective diagnostic and therapeutic options now available.^5^ To date, the hepatitis movement has been connected more to TB, malaria and other infectious diseases, but it does provide a model of goals, strategies and tactics for advocates in NASH. And I believe we have a responsibility to create a platform for all 500 million patients with liver disease and of course, the entire world population at risk.

## 6 CONCLUSION

Patients and committed caregivers have been the engines behind every major disease movement from Mary Lasker and Nancy Brinker in cancer to Jerome Stone in Alzheimer's to Martin Delany in HIV/AIDs and countless others, setting the agenda; organizing, funding and inspiring researchers; demanding the regulators and policymakers respond to the public interest by creating the infrastructure and processes that facilitate sustainable change. NASH is no different. Together, our results—breakthrough treatments, public empathy and empowered communities— can also be the same.
